# Randomized Clinical Trial: The Clinical Effects of Herb-Partitioned Moxibustion in Patients with Diarrhoea-Predominant Irritable Bowel Syndrome

**DOI:** 10.1155/2013/605460

**Published:** 2013-12-19

**Authors:** Yu-xia Ma, Xiao Liu, Cun-zhi Liu, Lin-peng Wang, Gang Guo, Dong-qing Du, Zhi-lei Wang, Hong Ma, Ping Qi, Zhao-feng Li, Yan-ping Guo, Hua-qiang Yi, Shu-zhong Gao

**Affiliations:** ^1^Shandong University of Traditional Chinese Medicine, Shandong, Jinan 250014, China; ^2^Beijing Hospital of Traditional Chinese Medicine Affiliated to Capital Medicine University, Beijing 10010, China; ^3^Qilu Hospital Affiliated to Shandong University, Shandong, Jinan 250012, China; ^4^Qianfoshan Hospital Affiliated to Shandong University, Shandong, Jinan 250014, China; ^5^Shandong University of Traditional Chinese Medicine, University Science Park, Chang-Qing District, Jinan 250355, China

## Abstract

*Objective*. To explore the efficacy of Herb-partitioned moxibustion in treating IBS-D patients. *Method*. 210 IBS-D patients were randomly assigned on a 3 : 3 : 2 basis to group HM, group FM, or group PB for 4-week treatment. The change of GSRS total score at weeks 4 and 8, the changes of GSRS specific scores, and adverse events were evaluated. *Results*. Patients in group HM and group FM had lower GSRS total score at week 4 (1.98 ± 0.303, 2.93 ± 0.302 versus 3.73 ± 0.449) and at week 8 (2.75 ± 0.306, 3.56 ± 0.329 versus 4.39 ± 2.48) as compared with patients' score in group PB. However, there was no significant difference of GSRS total score between group HM and group FM. The effect of HM was significantly greater than that of orally taking PB in ameliorating the symptoms of rugitus (0.38 versus 0.59, *P* < 0.05), abdominal pain (0.28 versus 0.57, *P* < 0.01), abdominal distension (0.4 versus 0.7, *P* < 0.01), and increased passage of stools (0.06 versus 0.25, *P* < 0.01) at the end of treatment period. In the follow-up period, patients' therapeutic effect in group HM remained greater than that in group FM (in abdominal pain, abdominal distension, and increased passage of stools) and that in group PB (in loose stools). *Conclusions*. HM appears to be a promising, efficacious, and well-tolerated treatment for patients with IBS-D.

## 1. Introduction

Irritable bowel syndrome (IBS), which is defined as “abdominal pain or discomfort that occurs in association with altered bowel habits over a period of at least three months” [1], is a common gastrointestinal (GI) disorder. Symptoms of IBS include abdominal pain, changes in bowel habits (diarrhea, constipation, or both), bloating, and incomplete defecation [2]. Although IBS does not end up with the development of serious disease and associated mortality, it does have a significant negative impact on patients' quality of life and social functioning [3] and can increase healthcare costs [4]. Unfortunately, IBS remains incurable and conventional medicine only provides some relief for individual symptoms [5]. Nowadays standard available therapies for IBS-D include antispasmodics, antidiarrhoeals, and 5-hydroxytryptamine_3_ (5-HT3) receptor antagonist [6]. However, a series of systematic reviews conducted by the American College of Gastroenterology Task Force showed poor quality of evidence that certain antispasmodics and antidiarrheals can reduce the frequency of stools but cannot affect the overall symptoms of IBS. They also noted that 5HT_3_ and 5-hydroxytryptamine_4_ (5HT_4_) agonists carry a possible risk of ischemic colitis and cardiovascular events, respectively, which may limit their utility [1].

It is common for IBS patients to seek complementary and alternative medicine (CAM) to treat their annoying bowel symptoms [7], and the percentage of IBS patients who have used CAM is 20–35% [8, 9]. A recent systematic review and meta-analysis of acupuncture for IBS found that patients reported greater benefits from acupuncture than from pharmacological therapies [10]. Another comprehensive review recorded that Chinese herbal prescription can decrease visceral hypersensitivity of IBS patients [11]. Both of them indicated the superiority of traditional Chinese medicine (TCM) in treating IBS.

Moxibustion therapy, which is used to treat diseases not less than acupuncture in China, is one of the three major therapies just as important as acupuncture and traditional Chinese herbal medicine, but less attention is paid to moxibustion therapy than acupuncture nowadays. Herb-partitioned moxibustion (HM) is a kind of moxibustion therapy, and it is used for treating many diseases but seldom used for treating IBS before. Whether there is curative effect of HM in ameliorating IBS symptoms remains unknown. Consequently, the aim of present trial is to explore the potential of HM to attenuate the symptoms of IBS-D.

## 2. Methods

### 2.1. Study Design

**Figure 1:**
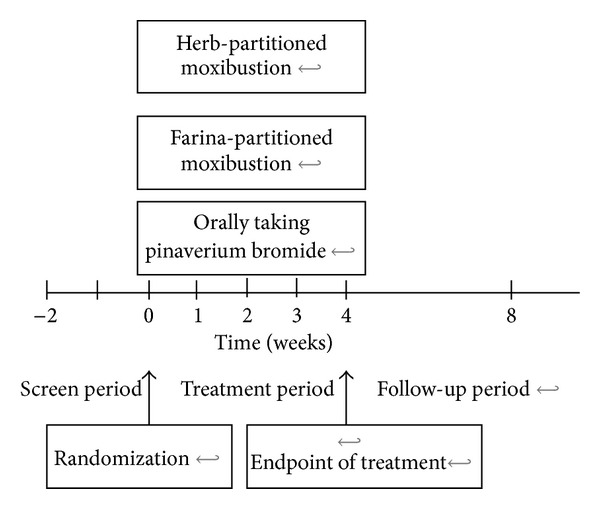
Schematic presentation of the study design.

The present study was a multicenter (3 centers), randomized, controlled trial (Figure 1).

#### 2.1.1. Patient Selection

Patients were recruited from the outpatients of Affiliated Hospital of Shandong University of Traditional Chinese Medicine, Qilu Hospital Affiliated to Shandong University and Beijing Hospital of Traditional Chinese Medicine Affiliated to Capital Medicine University, between October 2008 and January 2011.

Eligible patients met the following inclusion criteria: adult patients (aged 18–60 years) suffer from IBS-D as defined by Rome III [12] criteria and IBS-D was caused by spleen-qi deficiency according to the diagnostic methods of the Chinese medicine. At the end of the screen period, IBS-D patients were randomized into one of the three groups if they exhibited diarrhea the occurred for at least 2 days/week.

Patients excluded from study participation had the following: stool with pus and blood or with mucus; females who were pregnant or nursing; previous gastrointestinal or abdominal surgery; presence of primary disease such as cardiovascular disease, kidney disease, or hematopoietic system disease; diagnosis of any psychiatric disease; recent excessive consumption of alcohol, usage of any medication aimed for the treatment of IBS within the 2 weeks preceding randomization, or involvement in any investigational medications during the 2 weeks prior to screening. Patients who were allergic to the drugs used in the trial were also excluded.

#### 2.1.2. Randomization and Blinding

Randomization was performed by an independent statistician through generating allocation numbers based on a random number creation system. Eligible patients were randomly assigned on a 3 : 3 : 2 basis into herb-partitioned moxibustion group (group HM, herbs were used), farina-partitioned moxibustion group (group FM, farina was used as placebo for it contains no herb ingredients) or orally taking pinaverium bromide [13] group (group PB). The patients were randomly assigned in each center using a blocked randomization, and the block size is 16.

Double-blind could not be performed in this study. However, we can blind the patients between group HM and group FM. The evaluators, data collectors, and data statisticians were all blinded to treatment arm assignments.

The study protocol was approved by the Ethics Committee of Shandong University of Traditional Chinese Medicine Affiliated Hospital in 2010 (Registration no.: 20100110). All patients gave written informed consent. In order to insure the quality of this trial, treatment was performed by trained and certified clinicians who have the Chinese medicine practitioner license from the Ministry of Health of the People's Republic of China.

#### 2.1.3. Interventions

The medicinal herbs used in Group HM (seen in Table 1) were purchased from Jianlian Medicine Company (Jinan, China) and were kindly authenticated by Dr. Baoguo Li the professor of pharmacognosy (College of Pharmacy, Shandong University of Traditional Chinese Medicine, Jinan, China). The voucher specimens were deposited in Shandong University of Traditional Chinese Medicine. The voucher specimen number of the herbs was 080916.

The herbs were mixed in proportions (seen Table 1) and were shattered into medicamental pulverata by pulverizer. In group HM and group FM, the patients' navel and its surrounding area were disinfected using 75% alcohol. A bowl made by dough with a hole (diameter 2 cm, depth 2 cm) in the middle was placed on patient' navel. Musk and medicamental pulverata (about 8–10 g) were filled in the hole. The medicamental pulverata was replaced by farina (only contained flour, about 8–10 g) in group FM. Then a burning moxa cone (diameter 2 cm, height 2 cm) was put on the medicamental pulverata and changed till it burned out. Ten moxa cones were used during each treatment time. At the end of the treatment, the medicamental pulverata was sealed with adhesive tape and was washed clean 2 days later. The treatment (both in group HM and group FM) was performed twice a week and lasted for 4 weeks (treatment period). Patients in group PB orally took pinaverium bromide 50 mg three times a day for 4 weeks.

### 2.2. Efficacy Endpoints

#### 2.2.1. Primary Efficacy Endpoint

The primary efficacy endpoint is the total score of gastrointestinal symptom rating Scale [14] (GSRS total score). The GSRS was originally created and validated in Swedish [14, 15] for the assessment of GI symptoms. It contains seven items (abdominal pain, rugitus, abdominal distension, increased flatus, increased passage of stools, loose stools, and urgent need for defecation). In the present study, it was translated into Chinese and modified according to clinical practice. In this scale, each item was scored 0 (absence of the symptom), 1 (mild symptom), 2 (moderate symptom), or 3 (extreme degree of the symptom), rendering a total score between 0 and 21. The higher the score is, the more severe the symptom is.

#### 2.2.2. Secondary Efficacy Endpoint

Secondary efficacy endpoints are the GSRS symptom specific scores of abdominal pain, rugitus, abdominal distension, increased flatus, increased passage of stools, loose stools, and urgent need for defecation. The occurrence (number of times and frequency) and severity of all these symptoms with regard to GSRS were recorded in a diary by the patients themselves. Adverse events (AEs) were also recorded by clinicians during the treatment period, such as skin burn related to moxibustion therapy (the dropped ashes from the burning moxa cone) and infection caused by burn or allergy caused by the medicamental pulverata. All of them were recorded in detail throughout the study.

To insure the safety of this study, patients also received standard heart, renal, liver function laboratory tests and routine examination of blood, urine, and stools in the screen period and at the end of the treatment period (week 4).

Patients were followed up one month after the treatment (at week 8) by investigators using phone, mail, or e-mail.

### 2.3. Statistical Analysis

In the present study, the efficacy analyses were primarily performed on the full analysis set (FAS) corresponding to the intention-to-treat (ITT) population which included all randomized patients and secondarily on the per protocol (PP) population (including eligible patients who completed the whole study treatment).

The primary efficacy variable was the changes from baseline in GSRS total score. As for secondary endpoints, the changes of the GSRS symptom specific scores from baseline were analyzed. All these variables, both between-group and within-group comparisons, were made for exploratory advantage of curative effect.

We used Wilcoxon rank-sum test in between-group comparisons while the Wilcoxon's sign rank test was used in within-group comparisons. *P* values reported in this paper are two-sided and *P* values of <0.05 were considered statistically significant. All statistical analyses were carried out by using the Statistical Analysis System (SAS) version 8.1 (SAS Institute, Inc., Cary, NC, USA).

## 3. Results

### 3.1. Baseline Patient Characteristics

In our study, patients were mostly females (74.29%) and had a mean age of 25.7 years. GSRS total scores were similar among three different groups at baseline. And there were also no significant differences in GSRS symptom specific scores among three groups. The baseline age, gender, race, and the severity of IBS-D symptoms of the patients were listed in Table 2 and they were all similar among different groups.

**Figure 2:**
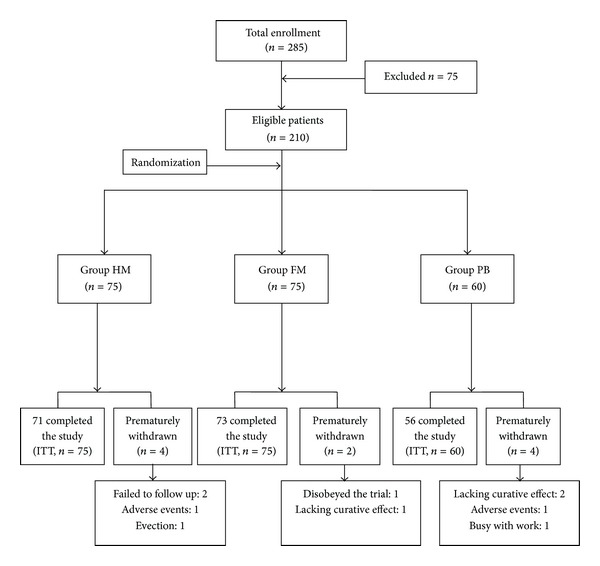
Flow chart of the trial. HM: herb-partitioned moxibustion; FM: farina-partitioned moxibustion; PB: orally taking pinaverium bromide; ITT: intention-to-treat.

In the present study, 285 patients were recruited, and 75 patients were excluded because they did not meet the inclusion criteria. In total, 210 patients (FAS) were randomized. The majority of randomized patients (*n* = 200, 95.24%, PP) completed the trials, whereas 10 (4.76%) patients discontinued the study. The most common reasons for study discontinuation were the lack of efficacy in three patients (group FM: one patient; group PB: two patients) and AEs in two patients (group HM: one patient; group PB: one patient). Patients' disposition and the size of the analysis population were summarized in Figure 2. Similar analytic results of PP population and FAS population were detected. Here we are going to introduce the analytic results of FAS population.

### 3.2. Primary Endpoint

**Figure 3:**
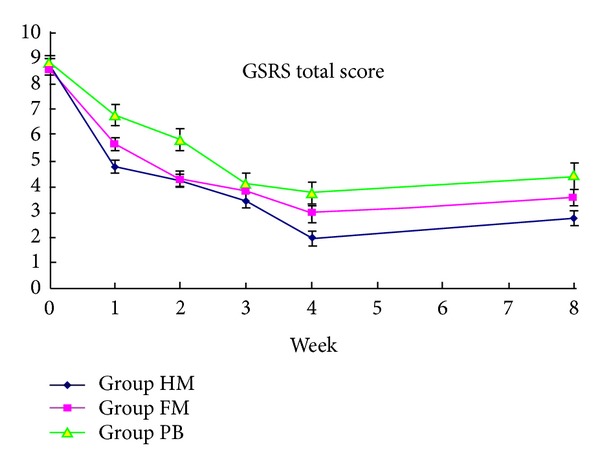
Primary endpoint: changes of the GSRS total score from baseline to week 4 (end of the treatment) and week 8 (follow-up period) for the three groups in the FAS population. HM: herb-partitioned moxibustion; FM: farina-partitioned moxibustion; PB, orally taking pinaverium bromide.

The GI symptom severity, as measured by the GSRS total score, was compared within-group and between-group. A decline in GI symptoms (GSRS total score) was identified throughout the treatment period in all the three groups (*P* < 0.01) (Figure 3). Within-group comparison between week 8 and week 4 (Table 3) showed no significant change of GI symptom in Group HM. However, in group FM (3.56 ± 0.329 versus 2.93 ± 0.302, *P* < 0.01) and group PB (4.39 ± 0.482 versus 3.73 ± 0.449, *P* < 0.05) the GI symptom was heavier at week 8 than at week 4. Between-group comparison (Table 3) showed that the relief of GI symptom was better in group HM than that in group PB at week 4 (−6.77 versus −5.12, *P* < 0.01) and at week 8 (−6.00 versus −4.46, *P* < 0.01), whereas no significant difference was found between group HM and group FM.

### 3.3. Secondary Endpoint

**Figure 4:**
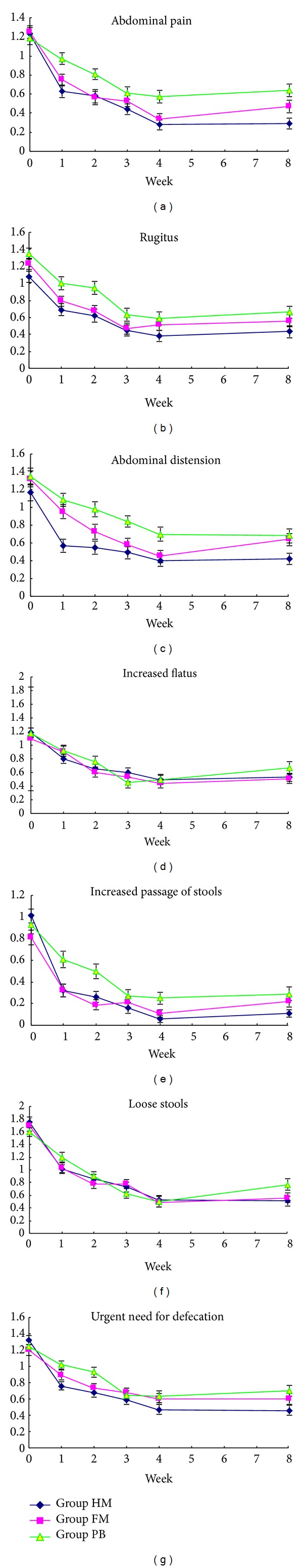
Secondary endpoint changes in GSRS specific score. (a) Abdominal pain; (b) rugitus; (c) abdominal distension; (d) increased flatus; (e) increased passage of stools; (f) loose stools; (g) urgent need for defecation during study time. HM: herb-partitioned moxibustion; FM: farina-partitioned moxibustion; PB: orally taking pinaverium bromide.

Figure 4 shows the changes in GSRS score for all seven IBS specific symptoms throughout the study time. There was significant relief of IBS specific symptoms from baseline to 4 and 8 weeks in all of the groups (*P* < 0.01). In group HM, the severity of all IBS specific symptoms was similar between week 4 and week 8. However, in group FM, IBS specific symptoms of abdominal pain, abdominal distension, and increased passage of stools were more serious (*P* < 0.05) from weeks 4 to 8. In group PB, IBS specific symptom of loose stools was more serious (*P* < 0.01) at week 8.

The comparison of effectiveness among three groups showed that at week 4, there was more relief of symptoms of rugitus (*P* < 0.05), abdominal pain, abdominal distension and increased passage of stools (*P* < 0.01) were seen in group HM compared to group PB. No difference was identified between group HM and group FM. At week 8, patients' symptoms of abdominal pain and abdominal distension were lighter (*P* < 0.05) in group HM than in group FM. Patients' symptoms of abdominal distension, loose stools (*P* < 0.05), abdominal pain, rugitus, increased passage of stools, and urgent need for defecation (*P* < 0.01) were lighter in group HM than in group PB.

### 3.4. Safety and Tolerability

No serious adverse events were reported during the whole study period. Only 1 patient (1.3%) in Group HM and 1 patient (1.7%) in group PB reported the allergy. Both of them were mild-to-moderate and were considered to be related to the study treatment. When we stopped the treatment on these two patients, the symptom of allergy disappeared. No clinically significant differences among the three patient groups were detected in the analysis of laboratory values, vital signs, and physical examination either.

## 4. Discussion

So far, the present study is the largest multicenter (3 centers) RCT with HM in IBS-D. Our results demonstrated that herb-partitioned moxibustion should be significantly better than orally taking pinaverium bromide in improving the GI symptoms of IBS-D. However, there was no significant difference between herb-partitioned moxibustion and farina-partitioned moxibustion. Additionally, the use of the HM was well tolerated and free from serious adverse effects. Only two patients were found to have light allergy during the study time and recovered without treatment.

Our findings are consistent with the previous clinical trial [16] including 81 patients. In that trial, HM was more effective than pharmacological therapy (responder rate 92.7% versus 62.5%) in ameliorating IBS symptoms. The animal experiment [17] reported that HM could decrease visceral hypersensitivity of rats. Importantly, our study found that the improvement of IBS symptoms in patients receiving HM and FM is superior to that of patients receiving pinaverium bromide in contrast to the studies 16 and 17 mentioned above, which did not have a FM control (or moxibustion alone) arm. This indicated that moxibustoin on navel (Shenque CV8) is better than orally taking PB in ameliorating GI symptom of IBS-D patients. Additionally, we found that there was no significant difference (*P* = 0.45) of patients' GSRS scores between group HM and group FM at weeks 4, which indicated that the warmth of burned moxa cylinder played a more important role than herbs in improving GI symptom of IBS-D patients. What is more, compared to week 4, GSRS specific score in Group HM remained stable in the follow-up period (week 8). However, the rebounding of several IBS-D symptoms was seen in Group FM and Group PB. This indicated that the herbs played an important role in the maintenance of curative effect.

HM is one kind of moxibustion therapies, which combines all the functions of moxibustion, acupoints and herbs. The moxa cylinder is burned to warm up herb-partition to stimulate acupoints instead of acupuncture needle, accelerating the penetration of the herbs into human body. Navel (Shenque CV8) is closed to large intestine and small intestine in anatomical position. What is more, it is also associated with spleen, large intestine, and small intestine according to meridian theory. And it is easy for the effective constituent of herbs penetrating into body due to the thin skin around it. In addition, the herbs used in our study were aimed to ameliorate symptoms of IBS-D in different aspects. Some of them are characterized with the nature of aromatic and warm character, such as Dingxiang and Shexiang, which can motivate the penetration of herbs. The remaining herbs were used to either reinforce spleen-qi or check diarrhea.

The present study plays an important role in the development of safer effective alternatives for the management of IBS-D there is significant unmet clinical need. However, it had some limitations. First of all, the potential limitation of the study is that, as a pilot study, no power analysis was provided, which lowered the statistical power of the study to a certain extent. Additionally, in order to maintain the compliance of the patients, 8 sessions of HM over 4 weeks were applied in the present study. But it may have been insufficient to achieve maximum effect from HM, because IBS-D is often a lifelong condition typified by chronic and episodic symptoms [6]. What is more, it would be helpful if the present study had a longer follow-up period to explore whether HM has a therapeutic approach that works for a long term. Finally, it is known that IBS-D had a severe impact on the quality of life [18–21] (QOL), which is correlated with the appearance of symptoms, the protracted time, and severity of the disease. Nevertheless, the impact of HM on IBS-D patients' quality of life was not analyzed in the present study, whereas many other studies [22–24] did. As a consequence, it is hoped that further studies can be carried out in this field.

## 5. Conclusion

The present trial provided preliminary evidence that HM may be a promising, efficacious, and well-tolerated treatment for IBS-D patients. This finding encourages further investigation of the efficacy of this method in larger, well-designed clinical trials. Here are some directions for future researches: optimal treatment duration and follow-up period should be determined to investigate the possible mechanism of action of HM, based on time, in treating with IBS-D. The impact of HM on IBS-D patients' overall satisfaction or QOL is needed, as well as the correlation between the dose of medicamental pulverata used in HM and the overall effect of HM therapy.

## Supplementary Material

The patient lies on his back and exposes the navel (Shenque, CV 8). A bowl made by dough with a hole (diameter 2 cm, depth 2 cm) in the middle was placed on patient' navel. The hole was filled with medicamental pulverata (about 8–10 g). The skin around the navel was covered by a towel with a hole in the middle to avoid skin burnt. Then a burning moxa cone (diameter 2 cm, height 2 cm) was put on the medicamental pulverata and changed till it burned out. Ten moxa cones were used during each treatment time.

## Figures and Tables

**Table 1:** Standard formula of ingredients of the medicamental pulverata.

Chinese name	Pharmaceutical name	Powered herb, %	Effect according to TCM
Bai Zhu	Atractylodis macrocephalae, rhizome	20	Invigorating spleen
Fu Ling	Poriae cocos, sclerotium (Hoelen)	15	Clearing damp and promoting diuresis
Ding Xiang	*Syzygium aromaticum *	15	Checking diarrhea
Shan Yao	*Dioscorea opposite *	10	Invigorating spleen-qi
Wu Bei Zi	Galla chinensis	40	Checking diarrhea
She Xiang	Moschus	A few	Motivating the penetration of herbs

**Table 2:** Baseline demographic characteristics and GSRS total score (ITT) in three groups.

Parameter	Group HM (75)	Group FM (75)	Group PB (60)	Total (210)	*P* value
Gender					
Female (%)	55 (73.33)	57 (76.00)	44 (73.33)	156 (74.29)	0.91
Male (%)	20 (26.67)	18 (24.00)	16 (26.67)	54 (25.71)	
Age (years)					
Mean (min, max)	26.69 (18.00, 59.00)	25.39 (19.00, 59.00)	24.97 (19.00, 60.00)		0.21
S.E.	1.045	0.908	1.012		
Race					
Han nationality (%)	74 (98.67)	73 (97.33)	60 (100.00)	207 (98.57)	0.43
Other nationalities (%)	1 (1.33)	2 (2.67)	0 (0)	3 (1.43)	
Marriage					
Married (%)	13 (17.33)	9 (12.00)	10 (16.67)	32 (15.24)	0.50
Single (%)	62 (82.67)	65 (86.67)	50 (83.33)	177 (84.28)	
Other (%)	0 (0)	1 (1.33)	0 (0)	1 (0.48)	
Course of disease (month)					
Mean (min, max)	63.49 (3.00, 480.00)	66.49 (3.00, 486)	42.67 (3.00, 360.00)		0.06
S.E.	8.231	10.947	6.859		
Combination of other drugs					
Yes (%)	0 (0)	0 (0)	0 (0)	0 (0)	
No (%)	75 (100.00)	75 (100.00)	60 (100.00)	210 (100)	
GSRS total score					
(Mean ± S.E.)	8.75 ± 0.251	8.60 ± 0.276	8.85 ± 0.287		0.71

S.E: standard error; GSRS: gastrointestinal symptom rating scale; ITT: intention-to-treat; HM: herb-partitioned moxibustion; FM: farina-partitioned moxibustion; PB: orally taking pinaverium bromide.

**Table 3:** Changes of GSRS total score in the three groups.

	Baseline (week 0) (mean ± S.E.)	Week 4 (mean ± S.E.)	Change at week 4	*P* value	Week 8 (mean ± S.E.)	Change at week 8	*P* value
Group HM	8.75 ± 0.251	1.98 ± 0.303	−6.77^∆^	<0.01	2.75 ± 0.306	−6.00^#^	<0.01
Group FM	8.60 ± 0.276	2.93 ± 0.302	−5.67	<0.01	3.56 ± 0.329*	−5.04	<0.01
Group PB	8.85 ± 0.287	3.73 ± 0.449	−5.12	<0.01	4.39 ± 0.482**	−4.46	<0.01

S.E.: standard error; compared with week 4, *P < 0.01, **P < 0.05; compared with Group PB, ^∆^ *P* < 0.01, ^#^ *P* < 0.01; GSRS: gastrointestinal symptom rating scale; HM: herb-partitioned moxibustion; FM: farina-partitioned moxibustion; PB: orally taking pinaverium bromide.
