# Supplementary figures and images for: Randomized Clinical Trial: The Clinical Effects of Herb-Partitioned Moxibustion in Patients with Diarrhoea-Predominant Irritable Bowel Syndrome
Source: Evid Based Complement Alternat Med. 2013 Dec 19;2013:605460. doi: 10.1155/2013/605460 (PMC3880695; doi:10.1155/2013/605460)

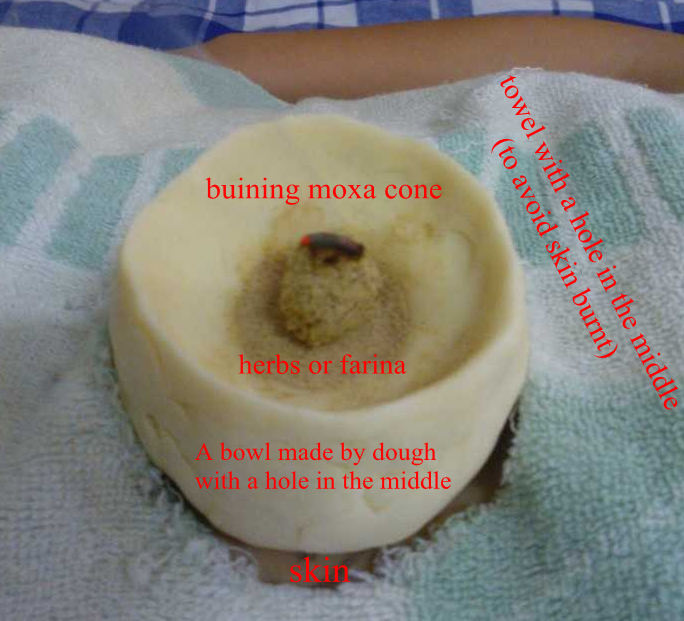

Supplement: Supplementary file 1 — The patient lies on his back and exposes the navel (Shenque, CV 8). A bowl made by dough with a hole (diameter 2 cm, depth 2 cm) in the middle was placed on patient' navel. The hole was filled with medicamental pulverata (about 8–10 g). The skin around the navel was covered by a towel with a hole in the middle to avoid skin burnt. Then a burning moxa cone (diameter 2 cm, height 2 cm) was put on the medicamental pulverata and changed till it burned out. Ten moxa cones were used during each treatment time. [file 605460.f1.doc]
